# Effects of the ketogenic diet on bone health: A systematic review

**DOI:** 10.3389/fendo.2023.1042744

**Published:** 2023-02-02

**Authors:** Vincenzo Garofalo, Federica Barbagallo, Rossella Cannarella, Aldo Eugenio Calogero, Sandro La Vignera, Rosita Angela Condorelli

**Affiliations:** Department of Clinical and Experimental Medicine, University of Catania, Catania, Italy

**Keywords:** ketogenic diet, low-calorie ketogenic diet, very-low-calorie ketogenic diet, bone health, osteoporosis, bone mineral density

## Abstract

**Objective:**

To carry out a systematic review of published studies to evaluate the relationship between different type of ketogenic diet (KD) and bone health as supported by the scientific literature.

**Methods:**

The study involved all articles that assessed the relationship between the use of KD for the treatment of overweight or obesity and bone health. The quality assessment was evaluated with using the Cambridge Quality Checklists. The search strategy included the following combination of Medical Subjects Headings terms and keywords: “osteoporosis”, ”bone health, ”bone function”, ”bone mineral density”, and “ketogenic diet”.

**Results:**

Seven trials were identified and reviewed. No significant changes in bone mass density (BMD) were observed after KD. The results showed no significant effect on bone resorption by measuring urinary N-telopeptide levels, on bone formation by measuring bone-specific alkaline phosphatase, or alterations in overall bone turnover in patients who followed KD. Only in female subject after a 10% weight loss, bone resorption increases while new bone synthesis decreases, but without increasing the risk of osteoporosis. Finally, patients on KD lost significantly more weight than controls, associated with an increase in serum vitamin D levels and a reduction in plasma parathyroid hormone (PTH) levels.

**Conclusion:**

No human studies have currently been conducted with adequate and powerful experimental designs to definitively understand the impact of KD therapy on bone health.

## 1 Introduction

Low-calorie ketogenic diet (LCKD) and very-low-calorie ketogenic diet (VLCKD) are diets low in carbohydrates and high in lipids, which have been shown to be effective in losing weight quickly and safely, as well as being able to improve body composition (1), athletic performance (2), and markers of cardiovascular and metabolic health (3). Over the last few years, KD has been widely accepted as an efficient method for the treatment of obesity and body weight management. The ketogenic diet is usually characterized by providing less than 20% of daily caloric intake as carbohydrates, more than 50% from lipids, and a moderate but variable amount of proteins (4). This type of distribution of macronutrients preserves glycogen and lean tissue protein utilization, increases fatty acid oxidation, and generates marked elevation of plasma ketone bodies (KB), such as acetate, acetone, and β-hydroxybutyrate (3βOH-B), known to be an effective alternative fuel source for tissues (5). Furthermore, β-hydroxybutyrate has been shown to have anti-inflammatory and anti-catabolic effects on skeletal muscle by inhibiting activation of the Nf-κB pathway (6). Several studies have shown that KD is effective in reducing body weight and fat mass, without inducing loss of muscle mass and fat-free mass (FFM), thus preventing the risk of sarcopenia (7, 8) and promoting the preservation of muscle strength (9). However, the preservation of muscle mass, known to be involved in glucose metabolism, in KD patients remains debatable.

Although the higher reduction in body fat and the cardiometabolic benefits, the use of low-carbohydrate diets has been associated with various adverse outcomes. KD causes alters vitamin D levels, lowers growth factors and a high “acid load” *via* the ketone bodies, these contribute to an increased risk for bone mineral density (BMD) loss (10). KD has previously been studied for its impact on bone mineral content (BMC), osteopenia, and osteoporosis, as well as common consequences related to this dietary treatment, such as hypercalciuria, urine acidification, and hypocitraturia (11). Evidence shows that this occurs due to the renal response, which consists of increased excretion of acid to compensate for the dietary acid overload. In turn, the skeleton acts as a buffer system and through its active resorption causes hypercalciuria and a negative effect on bone quality (12). Given the role of crosstalk between adipose tissue and bone, it is important to evaluate the effects of KD on bone metabolism and the possible mechanisms underlying the onset of osteopenia and osteoporosis.

Based on these premises, this study aimed to carry out a systematic review of published studies to evaluate the relationship between low-calorie and very-low-calorie ketogenic diet and bone health as supported by the scientific literature.

## 2 Methods

### 2.1 Search strategy

A systematic search was performed from January 2022 to November 2022, through Pubmed and Scopus databases from the earliest available date to November 2022, using Medical Subjects Headings (MeSH) indexes and keyword searches. The string used included the entry term “ketogenic diet*”, which was searched in combination (AND) with the terms “osteoporosis” OR “bone health” OR “bone function” OR “bone mineral density” OR “BMD”. Alternative entry term was “VLCKD”, “LCKD”, and “KD”. The query “LIMIT-TO (DOCTYPE, “ar”)” was used to limit retrieve only original studies. The same combination of terms was used for all the databases. The search strategy was performed in compliance with the Meta-Analysis and Systematic Reviews of Observational Studies (MOOSE) guidelines (13) (Supplementary Table 1) and the Preferred Reporting Items for Systematic Review and Meta-Analysis Protocols (PRISMA-P) (14) (Supplementary Table 2). Abstracts of the retrieved articles were independently screened by two researchers in duplicate (V.G. and F.B). Disagreements were resolved by a third person (R.A.C.).

### 2.2 Selection criteria

This systematic review included all published articles that evaluated the relationship between the use of KD for the treatment of overweight or obesity and bone health. All eligible studies were selected following the PICOS (Population, Intervention, Comparison/Comparator, Outcomes, Study design) (15) model (**Table 1**). Ketogenic diet consists of an extreme reduction in carbohydrate intake (<50 g/day) with a consequent increase in protein and fat intake. The aim of the ketogenic diet is to decrease appetite and increase lipolysis, resulting in increased use of fats as an energy source. There are different types of carbohydrate-restricted diets with varying protein and fat intake (16). Studies conducted on children treated with anti-epileptic drugs and athletes were excluded, given the impact on bone metabolism of epilepsy therapies and physical activity. Only articles in English reporting complete data of clinical relevance for the present review were included in the analysis. Duplicates have carefully been checked and removed.

**Table 1:** Inclusion and exclusion criteria of the current systematic review, according to the PICOS model (15).

	Inclusion	Exclusion
Population	Patients with obesity or overweight	Children, patients with hypogonadism, Cushing syndrome, hyperparathyroidism, renal failure, and other comorbilities capable of impacting on the bone density
Intervention	Ketogenic diet	Non ketogenic diets
Comparison	Non ketogenic diets (e.g. Mediterranean diet, fat diet) or no treatment	–
Outcome	BMD, UNTx, BSAP, PINP, β-Crosslaps, BMC	–
Study Type	Randomized controlled studies, case-control studies, cohort studies	*In vitro*, animal studies, case reports, editorials, communications, reviews, meta-analysis

BMC, bone mineral content; BMD, bone mineral density; BSAP, bone-specifc alkaline phosphatase; PICOS, Population, Intervention, Comparison/Comparator, Outcomes, Study design; PINP, procollagen type I N-propeptide; UNTx, urinary N-telopeptide.

### 2.3 Quality assessment

The quality of evidence (QoE) of the studies was evaluated by 3 investigators (VG, FB and RC), by using the Cambridge Quality Checklists (17).

## 3 Results

**Figure 1:**
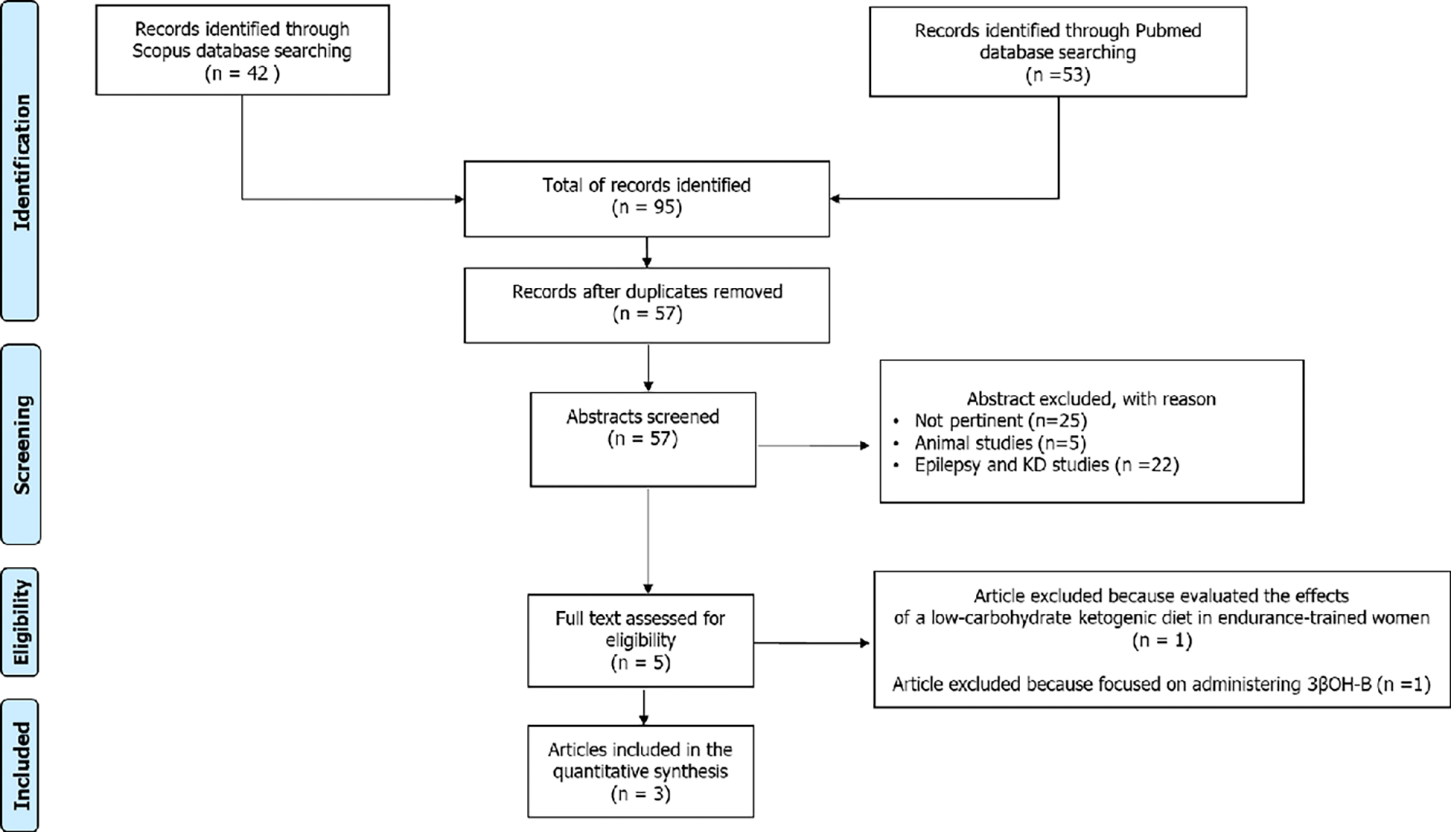
Study flowchart.

The aforementioned search strategy identified a total of 95 records. After the exclusion of 36 duplicates, the remaining 59 articles were considered potentially relevant for this review. After reading the abstracts, twenty-two articles were excluded because they were concerned about the correlation between epilepsy and ketogenic diet in children and adults, and 5 studies were conducted in animals. the remaining 23 articles were excluded as not pertinent. The full-text of the remaining 9 articles were downloaded and read carefully: two of these were excluded because one study evaluated the effects of a low-carbohydrate ketogenic diet in endurance-trained women and another focused on administering 3βOH-B. In conclusion, 7 studies were considered for this systematic review (**Figure 1**).

### 3.1 Quality of evidence of included studies

The 7 included studies were evaluated using the Cambridge quality checklist. Although this scale does not establish a precise threshold to differentiate between high- and low-quality studies, out of a total score of 15, five studies scored > 10, while only two studies scored 6 to 10 (**Table 2**).

**Table 2:** Quality of evidence assessment of the included studies [results of the **Cambridge Quality Checklist** (17)].

Study name	Type of study	Cambridge Quality Checklists		
		Checklist for correlates	Checklist for risk factors	Checklist for causal risk factors
Yu et al., 2022 (18)	CCT	2	3	3
Perissiou et al., 2020 (19)	RCT	3	3	7
Colica et al., 2017 (20)	RCT	4	3	7
Brinkworth et al., 2016 (21)	RCT	3	3	7
Foster et al., 2010 (22)	RCT	2	3	7
Carter et al., 2006 (23)	CCT	2	3	4
Jensen et al., 2001 (24)	RCT	3	3	7

CCT, Controlled Clinical Trial; RCT, randomized controlled trial.

### 3.2 Specific results

The main characteristics of the included studies are reported in **Table 3**. All of them evaluated the effects of KD on obese or overweight subjects. Among the included studies, all studies evaluated different parameters regarding body composition. Five studies (19–22, 24) used a dual-energy X-ray absorptiometry (DEXA) to assess bone mineral density (BMD) and bone mineral content (BMC). Two studies (18, 21) used β-Crosslaps, a collagen-degradation product that represents a biochemical marker of bone turnover. Only one study used different bone markers such as urinary N-telopeptide (UNTx) and bone-specific alkaline phosphatase (BSAP) to evaluate bone turnover in patients doing VLCKD, but only mean changes are reported (23). Procollagen type I N-propeptide (PINP) used to assess bone synthesis (18).

**Table 3:** Main characteristics of the studies included in the analysis.

	Type of study	Age (y)	Population (n)		BMI (Kg/mq)		Type of intervention	BMD (g/cm^2^)			
			Diet	Controls	Diet	Controls		Baseline		KD	
								Cases	Controls	Cases	Controls
Yu et al. 2022 (18)	CCT	35	75	NR	30.5	NR	LCKD	NR	NR	NR	NR
Perissiou et al. 2020 (19)	RCT	35 ± 9	31	33	31.2 ± 3	30.8 ± 4	VLCKD and prescribed exercise for 8 weeks	1.10 ± 0.05	1.12 ± 0.07	NR	NR
Colica et al 2017 (20)	RCT	45.4 ± 14.2	VLCKD1 = 20 VLCKD2 = 20	NR	30.45 ± 2.64	NR	Two types of VLCKD (with synthetic amino acids and with placebo) for 3 weeks	1.17 ± 0.19	1.19 ± 0.19	1.19 ± 0.18	1.19 ± 0.22
Brinkworth et al. 2016 (21)	RCT	51.6±6.5	32	33	33.7±4	33.2±4	LC diet for 52 weeks	1.26±0.10	1.26±0.09	1.22±0.09	1.23±0.08
Foster et al. 2010 (22)	RCT	46.2	153	154	36.1	36.1	LC diet for 104 weeks	1.1	1.1	0.00	0.00
Carter et al. 2006 (23)	CCT	40.8	13	13	33.51 ± 11.76	29.03 ± 3.88	LC diet for 12 weeks	NR	NR	NR	NR
Jensen et al. 2001 (24)	RCT	NR	62	NR	34	NR	LCKD with or without calcium supplement	NR	NR	NR	NR

BMD, bone mineral density; BMI, body mass index; CCT, controlled clinical trial; LC, very low carbohydrate diet; NR, not reported; RCT, randomised controlled trial; VLCKD, very low carbohydrate ketogenic diet.

All the seven studies showed a significant reduction in body mass index (BMI) after KD. No significant change in BMD and BMC was observed after KD (**Table 3**). Only one study observed a minimal decrease in total body BMD, but this decrease was not significantly different from the control (21), while patients who did not receive a calcium supplement during the diet had a BMC reduction (24). Two studies reported an increase in serum vitamin D levels (20, 24) and a decrease in HOMA index after KD. Regarding bone markers, no effect was reported neither on bone resorption [the mean UNTx decreased by 2.2 n/m (95% CI ± 27.2)], nor on bone formation [the mean BSAP decreased by 0.53 ug/l (95% CI ±2.96)]. Also, no alteration in overall bone turnover [the bone turnover ratio increased by 0.08 (95% CI ±0.81)] in patients who followed KD was described (23). In female subjects after a 10% weight loss PINP, the marker of bone synthesis, decreased remarkably (Δchange% 17.6%, p=0.000) and β-Crosslaps, the marker of bone absorption, increased remarkably (Δchange%−9.8%, p=0.035), but there was no increased risk of osteoporosis (18). Increase of β-Crosslaps has also been reported by Brinkworth et al. In two studies (18, 24) has been observed a significant decrease in serum parathyroid hormone (PTH).

## 4 Discussion

In the last two decades, the use of KD therapy has spread widely. The prevalence of some diseases, such as type 2 diabetes mellitus (T2DM), hypertension, dyslipidemia, sleep apnea, fatty liver disease, osteoarthritis, stress incontinence, gastroesophageal reflux, and polycystic ovary syndrome, could be reduced by weight loss (25). Specific metabolic disorders, in which KD is indicated in the clinical management of overweight/obese patients, have been listed by several guidelines, including that of the Italian Society of Endocrinology (26). Despite various hypotheses about the correlation between ketogenic diet (KD) and bone health in children, to date it remains unclear whether KD has any effect on bone health in adults. The considerations available are derived from studies conducted primarily in adults undergoing KD, similar to what was reported in children, although many of these studies do not provide relevant data on bone involvement. Clinical studies on the possible effects of KD on human bone health are poor and there are not many data on the long-term risk of osteoporosis in patients undergoing KD (26).

Although few studies on KD and skeletal metabolism are available, chronic metabolic acidosis is known to increase calcium excretion in the urine without increasing intestinal calcium absorption, leading to bone calcium loss by acute physicochemical dissolution and chronic increased bone resorption (27, 28). In an *in vitro* model of osteoblasts (OBL) cultures, the presence of certain types of ketone bodies affects different activities of alkaline phosphatase and mineralization. In particular, the mineralization activity of OBL appears to be upregulated by acetoacetate and downregulated by 3βOH-B (29). Therefore, all types of KDs that lead to metabolic acidosis could damage BMC. However, no studies have explored the effects of KD on bone health, and its does not lead to metabolic acidosis. Additionally, studies investigating the metabolic consequences of KD on calcium loss and bone health have not been conducted for longer than 3-4 months. Therefore, in patients using KD for prolonged periods or repeatedly in a cyclic manner for short periods, data on the impact of increased calcium loss on bone health are scarce.

Carter and colleagues published a study that evaluated whether a low-carbohydrate diet would lead to increased bone turnover in humans by measuring bone turnover markers. Thirty patients (15 undergoing a low-carbohydrate diet and 15 controls with no dietary restriction) were recruited for 3 months. The results showed no significant effect on bone resorption by measuring urinary N-telopeptide (UNTx) levels, on bone formation by measuring bone-specific alkaline phosphatase (BSAP), or alterations in overall bone turnover (BSAP/UNTx ratio) in patients who followed the low-carbohydrate diet for 1 and 3 months. No increase in bone turnover markers compared with controls was found in patients on the low-carbohydrate diet even though these patients lost significantly more weight than controls (23).

Other two markers of bone turnover, PINP and β-Crosslaps, show respectively a reduction and an increase in female subjects after loss of 10% body weight. The decrease in bone formation and increase in resorption during the initial phase of weight loss could be due to the rapid weight loss and energy restriction induced by KD, causing a mechanical unloading on the bone and consequently an increase in bone turnover, but without increasing the risk of developing osteoporosis (18).

A placebo randomized-controlled trial study in forty-two patients of both sexes analyzed the possible effects of two arms with different dietary treatments for three weeks each with a three-week washout interval. The two VLCKD treatments (<800 kcal/day) differed in terms of protein content and quality, specifically, in the first VLCKD1 arm, 50% of protein intake was replaced by synthetic amino acids, while in the VLCKD2 arm, a placebo was used. Before and after each dietary treatment, all patients were evaluated for various health parameters (health and nutritional status, anthropometric analysis, DXA-assessed body composition, bioimpedance metering, biochemical evaluation, and PPARγ expression by transcriptomic analysis), also including DXA-BMD and -BMC. After 21-days VLCKD, no negative changes were observed in global measurements of nutritional state including sarcopenia, BMC, BMD, liver, kidney, and lipid profile. In contrast, the left femur BMC was significantly increased after VLCKD1 (20). However, the DXA bone scans, at enrolment and the end of each dietary treatment, were not performed appropriately regarding the time interval (three weeks) between the two scans to best interpret the observed changes. In addition, considering the heterogeneity by age (ranging from 18 and 65 years) of the population analyzed, the BMD of the lumbar spine (LS) should have been reported as a Z-score (i.e., BMD normalized for BMD of persons of the same sex and age in the same population) and not as a T-score (i.e., BMD normalized for BMD of healthy young adults in the same population), given the presence in the study of men younger than 50 years and premenopausal women (30).

Combination of VLCKD and physical training with aerobic and resistance exercises result in significant improvement in the cardiometabolic profile of obese subjects (19). Combined exercise training may have also attenuated muscle mass loss, commonly observed with VLCKD. In addition, no changes were observed regarding BMD.

Obese patients have higher concentrations of the parathyroid hormone and lower blood concentrations of 25(OH)-Vit D than non-obese people, despite a higher habitual intake of vitamin D. Body mass index (BMI), fat mass, and waist circumference seems to be inversely correlated with levels of serum 25(OH)-Vit D, probably due to the large amount of adipose tissue, which can sequester this micronutrient, reducing its bioavailability. Buscemi and colleagues found that serum levels of 25(OH)-Vit D were inversely correlated with measures of general adiposity as BMI and fat mass size, suggesting that adipose tissue is an important influencing factor. Indeed, following VLCKD and subsequent weight/fat loss, a significant increase in 25(OH)-Vit D concentrations has been observed, this is in agreement with the hypothesis that in people with obesity, low 25(OH)-Vit D concentrations are due to its uptake and storage by adipose tissue, with subsequent release following fat mass reduction. In particular, the change in fat mass was correlated solely with the change in 25(OH)-Vit D blood concentrations, indicating the prominent role of this parameter as a possible depot (31).

Calcium supplementation during KD reduces BMC and urinary calcium loss, resulting in reduced PTH levels and thus reduced bone loss (24).

Foster et al. and Brinkworth et al. compared the effects of a low-carbohydrate diet and a low-fat diet on bone health (21, 22). A small reduction of BMD was observed in both diets, without a corresponding change in BMC. One possible explanation could be an artefact due to the instrument’s lack of sensitivity when weight and body composition vary and not a physiological change in BMD (32). An alternative explanation could be the longer duration of these studies compared to the others, which could lead to a greater loss of bone mass.

Studies on the effects of KD on the skeleton in adults are limited and are mainly conducted on narrow, specific, and particular populations, such as children with drug-resistant epilepsy. Several studies have explored the effect of KDs on skeletal development in children with epilepsy being treated with VLCKD. Bergqvist and colleagues demonstrated a reduction in BMC in children with epilepsy treated with KD, with follow-up after 15 months. The study protocol included questionnaires on daily calcium intake and assessment of BMC by DXA performed at a time interval appropriate enough for proper interpretation of the results (10). Combined treatment with anticonvulsant drugs and KD produces a greater degree of alteration in bone mineral metabolism than treatment with anticonvulsant drugs alone (33). AEDs appear to have a specific effect on the developing skeleton evidenced by the fact that epileptic adults treated with antiepileptic drugs (AEDs) since childhood have lower bone mass than epileptic adults who started AED therapy in adulthood (34).

Different studies in mice under KD treatment described low BMD and abnormal cancellous and cortical bone mass. Wu and colleagues showed that in mice the microarchitecture of the trabecular bone of the femur is impaired by KD to a level similar to that of ovariectomy (OVX). Measuring and comparing levels of tartrate-resistant acid phosphatase, to measure activities of osteoclasts, collagen type I, an early-stage marker of osteoblasts activity, and osteocalcin, a late-stage marker of osteoblasts activity, in the four groups, they found that the results found indicate that KD has a negative effect on trabecular and cortical bone quality in mice in a manner similar to OVX, in that both conditions result in a promotion of bone uptake through activation of osteoclasts rather than an inhibition of osteoblast-mediated bone formation (35). Another study demonstrated a significant decrease in the total BMD of rats fed KD for 12 weeks, with no difference in the serum calcium and phosphate concentration between the KD and control groups. Specifically, using micro-CT, it was observed that KD led to bone loss in cancellous and cortical bones (humerus and tibia), with insignificant changes in L4 vertebral bone. In addition, the stiffness and compressive strength of appendicular and axial bones decreased with KD and were highly correlated with the microstructural parameters of cancellous and cortical bones, as demonstrated by simulated compression analysis using micro-FE analysis (36).

An interesting study in mouse models evaluated the effects of administration of metformin, an oral antidiabetic drug, on KD + OVX-induced bone loss. The authors suggested that KD-induced cancellous bone loss is effectively attenuated using metformin while maintaining the biomechanical properties of long bones. However, further studies are needed to confirm the use of metformin as a potential treatment to prevent KD-induced osteoporosis in younger skeletons (37).

In conclusion, there are currently no human clinical studies with powerful and adequate experimental designs to definitively understand the impact of KD therapy on bone health. The few articles included in this systematic review showed no significant changes in bone metabolism in patients treated with KD. In children with intractable epilepsy, the combination of KD and AED could explain the reduction in BMD and bone mass. Animal studies show low BMD and abnormal cortical and cancellous bone mass, but these results have not been reported in human studies. Due to the lack of clinical studies on the impact of KD on bone health conducted in adult men and its long-term effects, it is not possible to determine whether KD can result in osteopenia and osteoporosis.

## Data availability statement

The raw data supporting the conclusions of this article will be made available by the authors, without undue reservation.

## Author contributions

Abstracts of the retrieved articles were independently screened by two researchers in duplicate: VG and FB. Disagreements were resolved by a third person: RC. The manuscript was written by VG, AC and SL. Materials and methods and results were developed by VG, FB and RC. Supervision of the manuscript and research work was done by RC. All authors contributed to the article and approved the submitted version.
